# Effectiveness of a Group-Based Progressive Strength Training in Primary Care to Improve the Recurrence of Low Back Pain Exacerbations and Function: A Randomised Trial

**DOI:** 10.3390/ijerph17228326

**Published:** 2020-11-11

**Authors:** Joaquín Calatayud, Benjamín Guzmán-González, Lars L. Andersen, Carlos Cruz-Montecinos, María Teresa Morell, Ricardo Roldán, Yasmín Ezzatvar, José Casaña

**Affiliations:** 1 Exercise Intervention for Health Research Group (EXINH-RG), Department of Physiotherapy, University of Valencia, 46010 Valencia, Spain; yasmin.ezzatvar@uv.es (Y.E.); jose.casana@uv.es (J.C.); 2 National Research Centre for the Working Environment, 2100 Copenhagen, Denmark; LLA@nfa.dk; 3 Laboratory of Clinical Biomechanics, Department of Physical Therapy, Faculty of Medicine, University of Chile, Santiago 8380419, Chile; benjamin.guzman08@gmail.com (B.G.-G.); ccmkine@gmail.com (C.C.-M.); 4 Sport Sciences, Department of Health Science and Technology, Aalborg University, 9220 Aalborg, Denmark; 5 Laboratory of Biomechanics and Kinesiology, San José Hospital, Santiago 8380453, Chile; 6 Primary Care Health Department Valencia Arnau-Llíria, 46015 Valencia, Spain; maite.cheste@gmail.com (M.T.M.); roldan_ric@gva.es (R.R.)

**Keywords:** core, chronic low back pain, multi-site, endurance, resistance training

## Abstract

Low back pain (LBP) is the leading cause of disability and one of the most common reasons for physician visits in primary care, with a 33% rate of recurrence during the first year. However, the most optimal exercise program in this context remains unknown. The objective was to evaluate the effectiveness of a group-based progressive strength training program in non-specific chronic LBP (CLBP) patients in primary care on pain recurrence and physical function. Eighty-five patients with non-specific CLBP were separated into two groups (Intervention group: completed a progressive strength training program 3 days per week for 8 weeks; Control group: received the usual care). The intervention group showed a recurrence rate of 8.3%, while the control group had a recurrence rate of 33.3% and a shorter time until the first recurrent episode. The intervention group showed increased lumbar extensor strength, left-hand handgrip strength, and reduced the number of pain sites compared with the control group. Results also showed greater odds for reducing LBP intensity and disability in the intervention group. In conclusion, a group-based progressive strength training program is a more effective and efficient alternative than Back-School programs and can easily be carried out in the primary health care context.

## 1. Introduction

Low back pain (LBP) is the leading cause of disability [1] and one of the most common reasons for physician visits in primary care [2]. Despite the fact that most episodes of LBP are short lasting, 33% of cases recur during the first year, converting LBP into a chronic condition (CLBP) [3]. Also, chronic widespread pain is highly prevalent among CLBP patients, which raises the risk of suffering from different comorbidities and psychosomatic symptoms [4]. Thus, CLBP is often accompanied by anxiety, depression, and poor quality of life [4]. All these factors translate into costs such as medical health care, transportation to appointments, work absenteeism, and productivity loss [5].

In primary health care services, there are no clear strength training recommendations for patients with CLBP. Back-School Programs focusing on education and exercise are the most common rehabilitation treatment [6] and even though physical exercise is recommended in CLBP [7], common primary care treatment lacks specific evidence based exercise guidelines, especially regarding dose and prescription [6]. In addition, strength training has demonstrated small but positive pain reductions [8], albeit no robust high quality evidence exists regarding their effectiveness over Back-School programs. While progressive strength training—i.e., progressing over time from low to high intensities—has been considered promising in the treatment of other musculoskeletal conditions [9], very few studies used this approach in CLBP, probably because of the inherent fear of heavy lifting while having LBP. A recent study [10] found that progressive elastic resistance training effects did not differ from general physical exercise at improving disability in patients with CLBP. However, the authors question the adherence to the home-based elastic resistance bands program since only 50% completed 60% of the sessions and some of them trained with lower intensities than prescribed [10]. Considering this, supervised group-based progressive strength training programs could enhance adherence and treatment effects by being performed under professional supervision and in social settings with other people [11].

Although there are many reasons for LBP, lumbar extensor deconditioning is closely associated with CLBP [12]. Characterized by spinal muscle atrophy [13], reduced lumbar extension strength/endurance and excessive fatigability of the lumbar extensors is significantly associated with the first development of LBP, with some cases leading to chronicity and disability [12]. In addition, gluteus maximus activity [14] and quality [15] have been found to be impaired in LBP patients, decreasing lumbar and sacroiliac joint stability, which could further impair the condition [16]. The latter contrasts with the actual evidence where few studies have considered comparing the effects of lumbar and hip extensors strengthening on the effects of usual primary care programs. While both exercise and Back-School programs have had positive effects on lumbar muscle strength [17], overall, exercise programs on their own is slightly preferred. Recent reviews concluded that physical exercise can prevent LBP [18] and its recurrence [19], however which treatment can prevent pain recurrence more effectively in primary care needs further investigation.

In this sense, progressive strength training could be optimal to restore lumbar deconditioning due to the gradual overload that firstly ensures muscle endurance adaptations with lighter intensities [20,21] and greater maximal strength [20,21] and neural adaptations [20] with later heavier intensities, with both eliciting similar muscle hypertrophy [20,21]. Furthermore, combining dynamic multi-joint and isometric exercises could be a better approach than simply selecting single-joint exercises. Dynamic multi-joint exercises are easy to quantify and more related to daily life activities [22], providing high muscle activity for several different muscles [23], and are also optimal for maximal strength gains [22]. In addition, isometric stabilization exercises can provide greater time under tension, with greater trunk stiffness gains than traditional dynamic core exercises [24].

The aim of this study was to evaluate the effectiveness of a group-based progressive strength training program in CLBP patients in primary care services to prevent pain exacerbation recurrence, strengthen lumbar extensor muscles, reduce back and widespread pain, and reduce disability. We hypothesized that the progressive strength training would result in significant reductions of LBP intensity and exacerbation recurrence and greater physical function when compared to usual primary care (Back School program).

## 2. Material and Methods

### 2.1. Participants

Inclusion criteria were having non-specific CLBP, age 18–75, living in the hospital area (Hospital Arnau de Vilanova, Valencia, Spain), and scheduled for rehabilitation in primary care. Subjects were excluded if: they had a severe somatic condition (e.g., cancer), psychiatric alteration, neurological disease, had or were waiting for spine surgery, or had participated in a similar exercise program on a regular basis during the last 6 months or had contraindications for high-intensity resistance training. All subjects who accomplished the inclusion criteria (assessed by a physician) were asked to participate. All participants were informed about the purpose and content of the project and gave their written informed consent to participate in the study. In addition, at the first follow-up, subjects had to give additional permission to use their register data at the hospital medical record for the second follow-up period. All procedures described in this section were approved by the institution’s review board (ethical committee approval number: H1520588795321) and comply with the requirements listed in the 1975 Declaration of Helsinki and its amendment in 2008. The study was registered in ClinicalTrials.gov (NCT03172962) and we adhered to the CONSORT guidelines to ensure transparent and standardized reporting of trials.

### 2.2. Procedures

#### 2.2.1. Randomization and Allocation

Participants were stratified by age and successively randomized during the study period to either intervention (progressive training) or control (usual Back-School program) following simple randomization procedures (computerized random numbers). The allocation sequence was performed by a second person and concealed from the main researcher supervising the training sessions.

#### 2.2.2. Intervention Group

**Figure 1:**
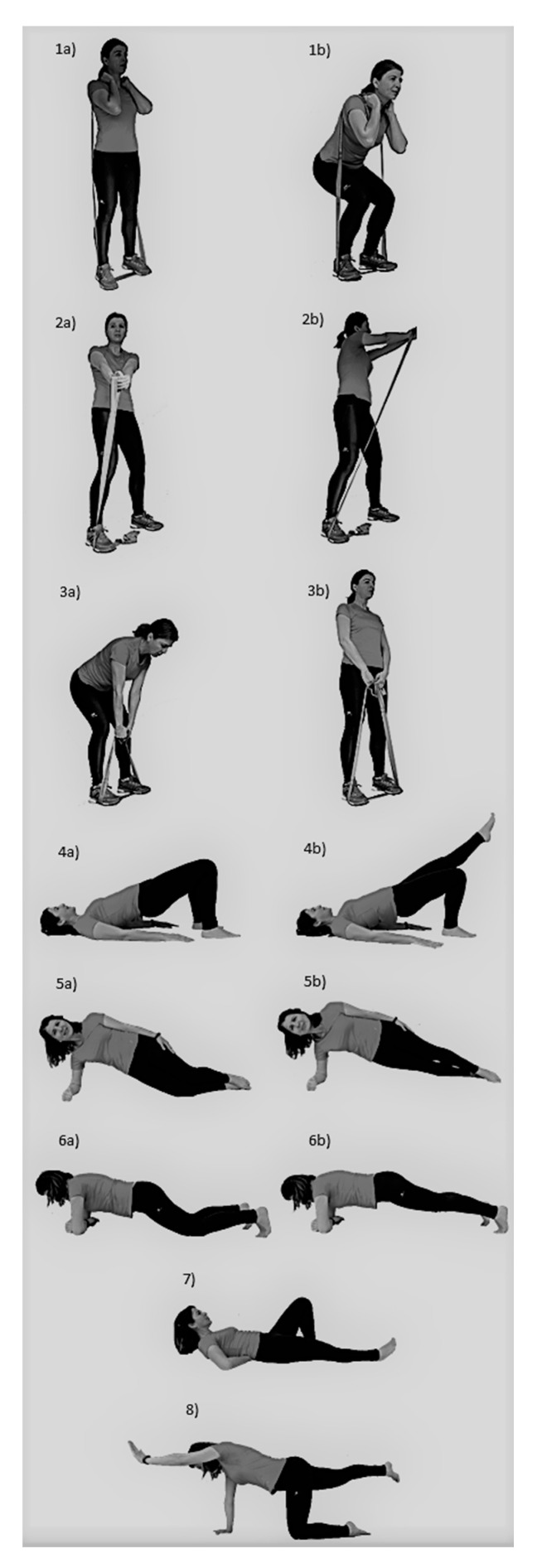
Exercises from the progressive strength training program. (**1a**) Squat initial phase; (**1b**) Squat final phase; (**2a**) Torso twist initial phase; (**2b**) Torso twist final phase; (**3a**) Deadlift initial phase; (**3b**) Deadlift final phase; (**4a**) Supine plank basic; (**4b**) Supine plank progression; (**5a**) Lateral plank basic; (**5b**) Lateral plank progression; (**6a**) Front plank basic; (**6b**) Front plank progression; (**7**) Modified curl up; (**8**) Bird-dog.

The progressive strength training program consisted of group-based training, especially focused on increasing core muscle strength (Figure 1) (Table 1). To avoid decreased personalized care in group-based interventions, the initial intensity and further progressions were individualized for each subject. To ensure adequate activity for the respective muscles, exercise selection was based on previous studies [25].

#### 2.2.3. Control Group

Participants in the control group performed the usual Back-School rehabilitation program for 8 weeks. Firstly, at the primary care center, subjects had 2 supervised sessions per week during the first 3 weeks in groups of 5–10 subjects. Afterward, subjects continued with the same protocol at home during the rest of the 5 weeks, performing the exercises daily.

**Figure 2:**
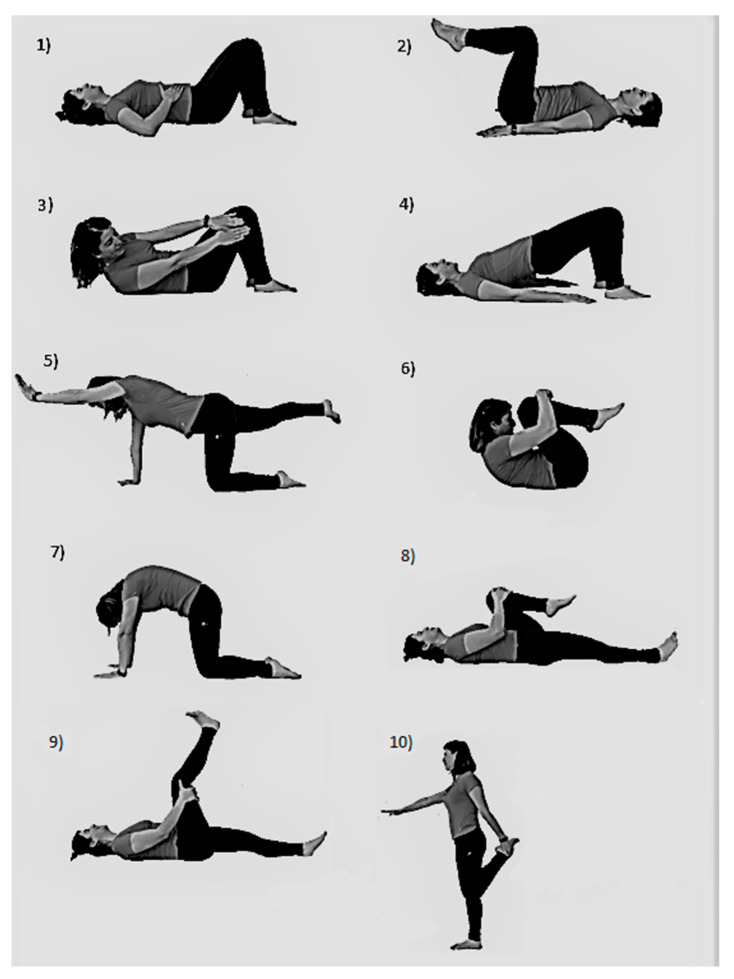
Back-School program exercises. (**1**) Abdominal hollowing; (**2**) Knee-up; (**3**) Oblique crunch; (**4**) Supine plank; (**5**) Bird-dog; (**6**) Knees-to-chest stretching; (**7**) Cat-camel; (**8**) Lying psoas stretching; (**9**) Lying hamstring stretching; (**10**) Standing quadriceps stretching.

The Back-School program was focused on performing 5 core strengthening exercises (abdominal hollowing, knee-up, oblique crunch, supine plank, bird-dog) and 5 stretching back and lower-limb exercises (knees to chest, cat-camel, lying psoas stretching, lying hamstring stretching, standing quadriceps stretching) (Figure 2). At the strengthening part, a set of 10 reps was performed for each exercise with a 3 s concentric contraction and 3 s eccentric contraction, with 10 s of rest between exercises. Each stretching exercise was maintained for 10 s and performed 4 times.

### 2.3. Outcomes

During the testing day, age, gender, weight, height, duration of current LBP, and leisure time physical activity were firstly recorded as descriptive data. The following variables were assessed:

#### 2.3.1. LBP Exacerbation Episodes

The first primary outcome was the between-group difference in the number of LBP exacerbation episodes (i.e., exacerbation recurrence) during the 100 days after the intervention. A follow-up of 100 days was considered appropriate to estimate the protective effect of the intervention, taking into account the detraining principle and that it seems logical that a better approach would be to not refrain from exercise. A secondary blinded person working at the hospital consulted this information at the hospital medical record. A pain exacerbation episode was defined as a pain increase during a day that disables the performance of daily life activities, resulting in professional management [26]. This was measured from each different person reporting an episode in primary care and not from the same person with several episodes. Also, the time frame until the first recurrence was assessed.

#### 2.3.2. LBP Intensity

The second primary outcome was LBP intensity. An 11-point numerical rating scale, where 0 = “no pain” and 10 = “the worst possible pain”, was used to assess the subject’s perception of LBP intensity during the last week. The numerical rating scale has an excellent test-retest reliability with an Intraclass Correlation Coefficient (ICC) of 0.83 [27].

In addition, the following secondary outcomes were assessed at baseline and after the completion of the program:

#### 2.3.3. Widespread Pain Sites

Subjects were asked to highlight painful sites in the last week at the Nordic Questionnaire drawing [28]. The total number of painful sites was recorded for later analysis.

#### 2.3.4. Analgesics

The number of days the participants used analgesics during the last week due to LBP was assessed using a questionnaire [11].

#### 2.3.5. Disability

The Roland-Morris Questionnaire was used to assess physical disability due to LBP. In a study conducted with patients with acute/subacute CLBP, the test-retest reliability showed ICC ranging from 0.42 to 0.53 [29].

#### 2.3.6. Isometric Lumbar Extension

The “Biering-Sorensen test” was used to assess the isometric endurance of trunk extensor muscles. The test was performed according to previous recommendations [30]. High test-retest reliability has been found in a recent meta-analysis for this test (ICC = 0.93–0.97), also with a good interrater reliability (ICC = 0.88–0.99) [31].

#### 2.3.7. Handgrip Strength

To assess this outcome, a TKK digital hand dynamometer (TKK 5101 Grip-D, Takey, Tokyo, Japan) was used. Subjects were standing with their arm straight down their side, with their shoulder slightly abducted (approximately 10°), elbow fully-extended, forearm in a neutral position, and wrist extended [32]. Subjects were asked not to touch any part of their body with the dynamometer except the hand being measured. A practice trial was performed and then 3 trials were performed with each hand, encouraging subjects to produce maximum force effort during 3 s. The highest value was used for the analysis. The TKK has showed high reliability with a very low systematic error (0.02) [32].

### 2.4. Statistical Analysis

Between-group differences from baseline to follow-up were calculated using linear mixed models (proc mixed). Analyses were performed using SAS statistical software (SAS version 9.4) (Statistical Analysis Software, Cary, NC, USA) according to the intention-to-treat principle, including all participants regardless of loss to follow-up. Analyses were adjusted for the baseline level of the outcome. The estimation method was restricted maximum likelihood (REML) with degrees of freedom based on the Kenward-Roger approximation. *p*-levels < 0.05 were accepted as statistically significant.

LBP exacerbation recurrence was treated as a binary outcome during a 100-day follow-up after the termination of the intervention and was compared between intervention and control using logistic regression (Proc Genmod, SAS version 9.4). Only those subjects who during the first follow-up permitted us to use their register data were included in this analysis.

Effect size (Cohen’s d) and % change were calculated. The effect size was described as: <0.2 = trivial effect; 0.2–0.5 = small effect; 0.5–0.8 = moderate effect; >0.8 = large effect. Minimal clinically important differences were calculated according to a previous study [33] by multiplying the pooled baseline standard deviation scores by 0.2.

To estimate the sample size, a clinically relevant difference between the groups of at least one standard deviation of the continuously distributed main variable within groups was used. Because a significance level of 5% and a power of 80% were used, at least 17 participants had to conclude the study in each group.

## 3. Results

**Figure 3:**
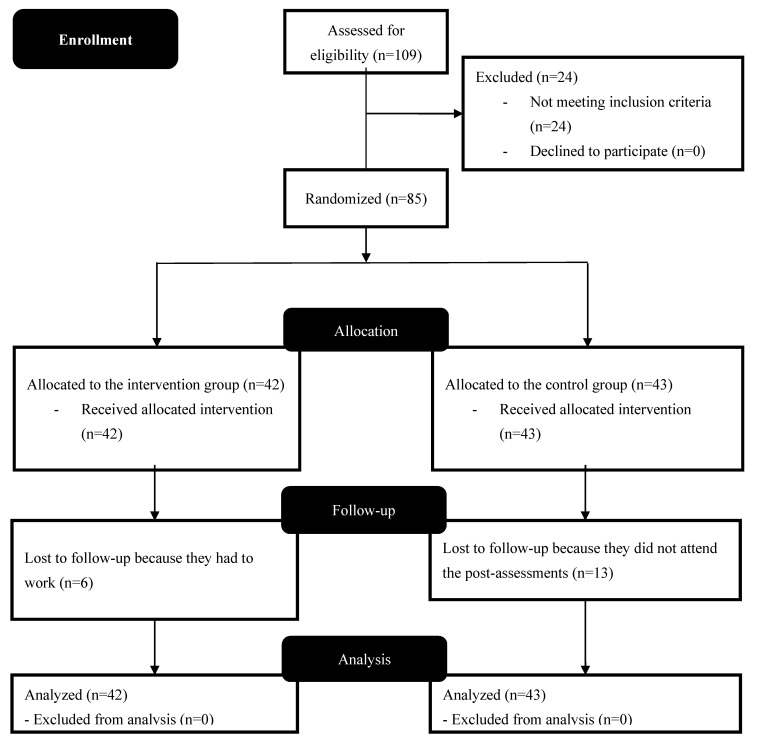
Flow diagram of the progress through the phases of the study.

Demographic data was as follows: the control group (*n* = 43) had a mean age of 50 ± 12 years, a height of 165 ± 7 cm, and a weight of 72 ± 14 kg. The intervention group (*n* = 42) had a mean age of 52 ± 11 years, a height of 164 ± 10 cm, and a weight of 76 ± 19 kg. Figure 3 shows the complete flow chart diagram of the progress through the phases of the study.

Table 2 shows LBP exacerbation recurrence episodes during the follow-up (primary outcome). The intervention group showed a recurrence rate of 8.3%, while the control group had an increased recurrence rate (33.3%) and a shorter time until the first recurrent episode.

Table 3 shows the results from the other primary and secondary outcomes. The intervention group had increased lumbar extensor strength, left-hand handgrip strength, and a reduced number of pain sites compared with the control group. Odds ratio estimates results showed greater odds for reducing LBP intensity (point estimate of 3.08; 95%CI 1.04–8.99; *p* < 0.0001) and disability (point estimate of 1.932; 95%CI 0.56–6.66; *p* < 0.0002) in the intervention group. Minimal clinically important differences were as follows: Isometric lumbar extension = 5.71; LBP intensity = 0.42; Number of pain sites = 0.48; Analgesics = 0.53; Handgrip strength left hand = 2.13; Handgrip strength right hand = 2.09; Disability = 1.08. All between-group differences were clinically important, except for Analgesics and Handgrip strength right hand.

## 4. Discussion

The aim of this study was to study the effects of a group-based progressive strength training program in CLBP patients in primary care services on pain exacerbation recurrence, lumbar extensor muscles strength, lower back and widespread pain, and disability. The main finding was the lower LBP exacerbation recurrence in the intervention group. Other relevant findings favoring the intervention were the large effect size when increasing the lumbar extension endurance and the moderate size effect when reducing multi-site pain zones. While the intervention did not cause a significant reduction in disability or LBP intensity compared to the Back-School Program, it did show a greater odds ratio for a clinically relevant improvement of both outcomes.

Our long-term follow-up showed lower LBP recurrence after the progressive resistance training (8.3%) compared with the Back-School program (33.3%), which behaved according to previous reports [3]. To our knowledge, this is the first study in primary care to compare in a randomized trial the effects on LBP recurrence of a standardized progressive strength training program against Back-School programs. Previous studies have also found similar recurrence reduction after other exercise programs for LBP at 100 days (approximately 5.9%) [26], which in some way are in line with findings at 6 months (approximately 8.8%) [26] and 12 months (32%) [26], whilst back school programs in primary care do not show the same effectiveness [26]. The lower recurrence of exacerbation may be partly explained by the large improvement the progressive strength training program had over the musculoskeletal system as found in the Biering-Sorensen test (from 35 s to 79 s; values below 58 s have been related with a twofold chance of having LBP [34]) and in the handgrip strength of the left hand. Since the left hand was the non-dominant hand in most of the participants, a greater opportunity window for improving strength at this side may be expected. Handgrip improvements may have some relevant functional consequences for basic daily life activities, especially for those who are older. In fact, low handgrip strength is predictive of mortality, longer hospital length of stay, and limited physical function [35].

The strength training group showed greater odds for a clinically relevant reduction of pain and disability, although when used as continuous variables, the intensity of these did not reach statistical significance. These results are somewhat in line with the findings of similar studies where significant improvements were seen for perceived pain and disability after progressive strength training [36]. Despite the common belief, a review found that lumbar physical function (i.e., lumbar muscle strength, endurance, and mobility) is not directly related to pain and disability after an exercise therapy for CLBP, arguing that the beneficial effects of exercise are more from the “central nervous system” and that disability should be aimed at by improving overall physical function and psychological factors rather than only local (i.e., lumbar) physical function [37]. Thus, the greater odds for pain and disability reduction in the intervention group may not be due to muscle strengthening by itself but also to the exercise therapy and its psychological effects. On the one side, group exercise therapy influences psychological factors such as psychological distress, fear-avoidance beliefs, catastrophizing, and coping strategies [38], improving their perception, and on the other side, muscle strengthening may result in enhanced lumbar and sacroiliac joint stability [16], therefore improved physical function. The improvements in strength, endurance, and pain perception, combined with the unharmful experiences while exercising, may stimulate subjects to gain trust in the function of their back and feel increased ability to perform activities of daily living such as transportation or dressing. In this sense, using group-based interventions focused on strengthening programs with progressive loading could be optimal, so while physiological adaptions occur as each exercise intensity step is completed, subjects can increase their self-efficacy and improve their perception of pain and disability.

The reduction of widespread pain was another relevant finding due to its clinical importance [4]. While the Back-School program group did not reduce the number of zones with pain, the intervention group decreased theirs by two. This is in line with previous studies where resistance training had effects on the main pain region and in additional ones [39]. As increased pain sensitivity may be one of the reasons for multi-site pain zones [40]. Our results suggest that the progressive resistance training program would decrease pain sensitization, as proposed by a previous study using resistance training to reduce the additional number of pain regions in neck cases [39].

While the 100-days follow-up period could be a limitation, it seems logical that future studies should have a greater focus on conducting longer interventions instead of evaluating long-term adaptations that will presumably be lost after refraining from exercise. Finally, care should be taken when extrapolating these results to other populations that are not able to perform additional exercise. In those settings, a different approach consisting of a very slowly introduced progressive exercise protocol and/or other interventions as behavioral therapy could be studied to ensure their effectiveness.

## 5. Conclusions

A group-based progressive strength training program can be applied in populations with low back pain with the aim to improve physical function and reduces recurrence and primary care visits more than the most frequently used primary care programs. Importantly, our program can easily be implemented in primary health care at a low cost and with minimum supervision for a group of patients.

## Figures and Tables

**Table 1:** Exercise program.

Frequency	3 days per week for 8 weeks.
Schedule	Sessions were performed at the same time of the day (i.e., during the morning) and were separated by 48 h (i.e., Monday, Wednesday, Friday).
Location	Sport facility in a primary care center.
Supervision	Sessions were supervised by a physical therapist, neither involved in the randomization nor in data collection.
Exercise order	In each session, the dynamic exercises were performed in a different order and in a circuit manner, switching from one exercise to the next so that the muscles were fatigued alternately and without rest between exercises. Secondly, five isometric plank exercises were performed.
Dynamic exercises	A warm up set was performed before each specific exercise by using light resistance to easily perform 10 repetitions without fatigue. Intensity progressively increased each two weeks, from 20 repetition-maximum (RM) to 10 RM (i.e., 20 RM, 15 RM, 12 RM, 10 RM). To achieve adequate exercise intensity during dynamic exercises, the elastic bands were pre stretched to approximately 50% of the initial length (initial length, 1.9 m) and then different bands were used/added when needed to reach the desirable intensity. For this purpose, red, blue, black, silver, and gold elastic band colors were available (TheraBand CLX, The Hygenic Corporation, Akron, OH, USA), alone or combined in parallel. Three sets of each exercise were performed. In these exercises, movement velocity was performed at a rate of approximately 1.5 s for concentric and 1.5 s for eccentric phases. In cases of pain, the intensity was reduced to the previous step or range of motion was restricted until pain decreased.
Isometric exercises	Intensity progression was based on reducing the base of support or focusing on activating the abdominal muscles. This progression was performed when subjects were able to do the basic exercise with the proper technique and during the required volume. In addition, training volume and thus total time under tension increased during the isometric exercises by progressively increasing the number of repetitions each two weeks: (1) 15 reps of 5 s (75 s total); (2) 20 reps of 5 s (100 s total); (3) 25 reps of 5 s (125 s total); (4) 30 reps of 5 s (150 s total). If subjects were not able to complete the exercise progression during the desired time due to pain, they had to return to the basic exercise.

**Table 2:** Low Back Pain (LBP) exacerbation recurrence episodes during the follow-up.

	n	Episodes	%	Relative Risk (95% Confidence Interval)	Chi-Square	*p*-value	Mean Days until First Recurrence Episode
Control	30	10	33.3	4 (1.2–13.2)	5.2	0.02	57.8
Intervention	36	3	8.3				62.7

**Table 3:** Results from the other primary and secondary outcomes (*n* = 85). SD = Standard Deviation; CI = Confidence Interval.

	Control		Intervention		Between-group Difference at Post			*p*-Value (Post)	Effect Size	% Change Control Group	% Change Intervention Group
					Mean	95% CI					
	Mean	SD	Mean	SD		Lower	Upper				
Isometric Lumbar extension pre (s)	25.97	29.93	34.61	28.6	42.8	−61.4	−24.1	<0.001	1.50	14.25	128.26
Isometric Lumbar extension post (s)	29.67	28.06	79	58.19							
LBP intensity pre	6.3	2	6.2	2	0.8	−0.4	1.9	0.193	0.36	−19.05	−30.65
LBP intensity post	5.1	3	4.3	2							
Number of pain sites pre	3.2	2.2	4.4	2.5	1	0.1	1.7	0.030	0.42	−7.41	−54.95
Number of pain sites post	3.0	2.4	2.0	2.4							
Analgesics pre (days/week)	4.23	2.69	3.69	2.57	0.5	−0.5	1.5	0.339	0.19	−21.75	−33.33
Analgesics post (days/week)	3.31	2.97	2.46	2.67							
Handgrip left hand pre (Kg)	32.36	10.51	27.51	10.35	3.1	−5.7	−0.4	0.024	0.29	3.68	22.72
Handgrip left hand post (Kg)	33.55	10.6	33.76	12.39							
Handgrip right hand pre (Kg)	31.73	10.28	28.3	10.65	1.4	−3.9	1.1	0.259	0.14	3.47	13.25
Handgrip right hand post (Kg)	32.83	11.54	32.05	11.82							
Disability pre	10.2	5.52	7.75	5.08	1.6	−0.3	3.5	0.107	0.29	−22.55	−35.87
Disability post	7.9	5.35	4.97	4.2							
